# Cytokine and Lipid Mediator Regulation of Group 2 Innate Lymphoid Cells (ILC2s) in Human Allergic Airway Disease

**DOI:** 10.4172/2576-3881.1000116

**Published:** 2017-08-04

**Authors:** Kellen Cavagnero, Taylor A. Doherty

**Affiliations:** Department of Medicine, University of California San Diego, USA

**Keywords:** Group 2 innate lymphoid cells (ILC2), Type 2 cytokine, Leukotrienes, Asthma, Rhinitis, IL-33, TSLP, Lipid mediators, Allergic airway disease, Aspirin Exacerbated Respiratory Disease (AERD)

## Abstract

The recent discovery of group 2 innate lymphoid cells (ILC2s) has caused a paradigm shift in the understanding of allergic airway disease pathogenesis. Prior to the discovery of ILC2s, Th2 cells were largely thought to be the primary source of type 2 cytokines; however, activated ILC2s have since been shown to contribute significantly, and in some cases, dominantly to type 2 cytokine production. Since the discovery of ILC2s in 2010, many mediators have been shown to regulate their effector functions. Initial studies identified the epithelial derived cytokines IL-25, IL-33, and TSLP as activators of ILC2s, and recent studies have identified many additional cytokine and lipid mediators that are involved in ILC2 regulation. ILC2s and their mediators represent novel therapeutic targets for allergic airway diseases and intensive investigation is underway to better understand ILC2 biology and upstream and downstream pathways that lead to ILC2-driven airway pathology. In this review, we will focus on the cytokine and lipid mediators that regulate ILC2s in human allergic airway disease, as well as highlight newly discovered mediators of mouse ILC2s that may eventually translate to humans.

## Introduction

Group 2 innate lymphoid cells (ILC2s) are a relatively newly discovered lymphocyte population that promotes features of allergic airway diseases including asthma and chronic rhinosinusitis through secretion of the type 2 cytokines IL-4, IL-5 and IL-13 among others. Unlike Th2 cells, ILC2s lack T cell receptors and, therefore, are not controlled in an antigen-specific manner [1]. Additionally, ILC2s lack surface receptors expressed by the major hematopoietic lymphocyte lineages including B, T, natural killer T (NKT), and natural killer (NK) cells [1,2]. In mouse models of asthma, ILC2s have been shown to drive airway eosinophilia, hyperresponsiveness, and mucous hypersecretion that are all features of allergic asthma [1,2]. Recently, increased levels of ILC2s have been detected in patients with allergic airway diseases including asthma, allergic rhinitis (AR), and chronic rhinosinusitis (CRS) [3–12]. Further, ILC2s are recruited to nasal mucosa from the blood in aspirin exacerbated respiratory disease (AERD), which is characterized by intense eosinophilic nasal polyps, asthma and respiratory reactions after COX-1 inhibition [13,14].

Studies examining the regulation of ILC2 homeostasis, activation, and proliferation have identified a myriad of mediators that both promote and inhibit their function. Early ILC2 studies using mouse models of asthma with protease-derived allergens demonstrated that damaged airway epithelial cells release cytokines that include IL-25, IL-33, and thymic stromal lymphopoietin (TSLP), which directly and potently activate ILC2s, leading to production of IL-4, IL-5, IL-9, and IL-13 [1,2]. Interestingly, certain ILC2 activators are capable of inducing select type 2 cytokine production. For example, stimulation of ILC2s with IL-33 results in IL-5 and IL-13 – yet minimal IL-4 – production, whereas stimulation with lipids, such as cysteinyl leukotrienes (CysLTs) and prostaglandin D2, causes robust IL-4 production [15]. Furthermore, combinations of ILC2 activators that utilize non-redundant signaling pathways can result in additive and even synergistic activation [16].

**Figure 1:**
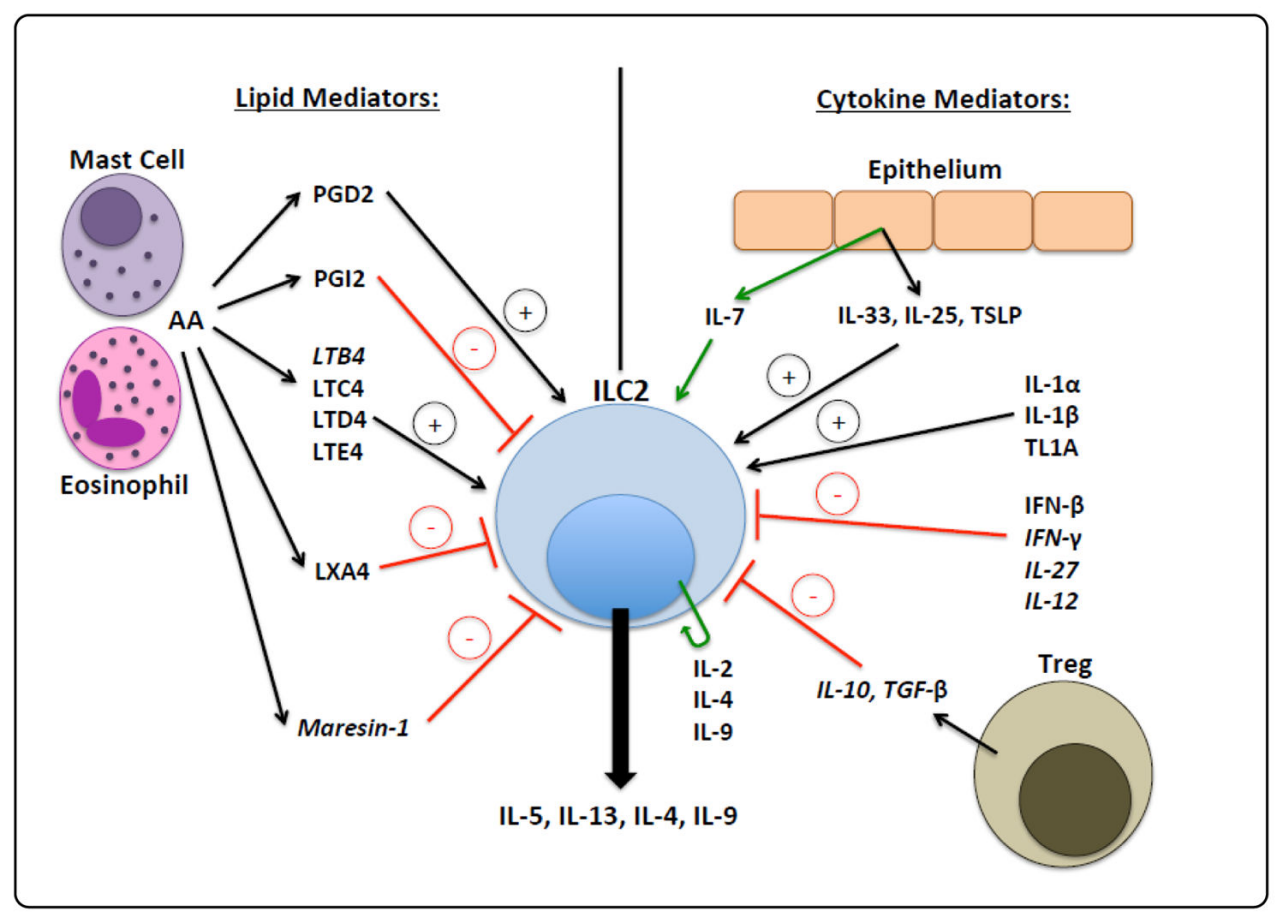
Lipids and cytokines that regulate ILC2s. Mediators only found in mouse are italicized and green lines represent maintenance/homeostatic cytokines.

Each Th2 cytokine downstream of ILC2s plays an important role in disease pathogenesis: IL-4 drives naïve T cell differentiation into Th2 cells and B cell IgE class switching, and supports ILC2 homeostasis; IL-5 drives airway eosinophilia; IL-9 stimulates ILC2 cell proliferation and inhibits apoptosis in an autocrine fashion; and IL-13 induces airway hyper responsiveness, goblet cell metaplasia, mucous hyper secretion, and IgE class switching [1,2]. This review will largely focus on the upstream cytokine and lipid mediators that regulate ILC2s in human allergic airway disease, which are summarized in Figure 1. In addition, we will highlight newly discovered mediators of mouse ILC2s that may also translate to humans.

## Epithelial Cytokines that Regulate ILC2s

### IL-33

IL-33 has been shown to be a potent driver of experimental allergic airway disease in mice, and its expression has been linked to many human allergic airway diseases [17]. In humans, IL-33 expression is highly upregulated in AERD patient nasal polyps, AR patient nasal secretions, and asthmatic airways (with severe asthmatics demonstrating the highest expression) [14,18,19]. Surprisingly, IL-33 expression is not upregulated in CRS polyps, though the IL-33 receptor has been detected at higher levels [19]. Importantly, large population genome wide association studies have shown that small nucleotide polymorphisms (SNPs) involving IL33 and its receptor are associated with increased asthma susceptibility [20].

IL-33 is classically considered an epithelial derived cytokine; however, endothelial cells, smooth muscle cells, and macrophages also express IL-33 [1,2,18]. IL-33 and IL-25, which is also epithelial cell-derived, were the first cytokines demonstrated to activate murine ILC2s [21]. In mice, IL-33 administration induces a robust type 2 immune response independent of adaptive immunity, which was one of the first clues to the presence of a novel non-T cell producing Th2 cytokines [2,22]. The IL-33 receptor is a heterodimeric receptor composed of ST2 and IL-1R (T1/ST2) [2]. Additional cells types including Th2 cells, eosinophils, dendritic cells (DCs), macrophages, basophils, mast cells, and NKT cells also express T1/ST2 and are activated by IL-33 [1,2]. In a landmark 2011 human study, ILC2s in peripheral blood and fetal gut were shown to produce IL-13 in response to IL-33 stimulation *in vitro* [9]. Importantly, the authors were the first to identify ILC2s in human lung tissue [9]. Overall, these findings suggest that the diseased human airway is an environment where IL-33 and/or its receptor are increased along with mucosal ILC2s. Thus, IL-33-induced activation of ILC2s very likely plays a role in human allergic airway disease pathogenesis.

### IL-25

Similar to IL-33, the epithelial cytokine IL-25 has been shown to promote experimental allergic airway disease in mice, and its expression has been linked to human allergic airway disease. Corrigan et al. found that human asthmatic bronchial biopsies from patients challenged with allergen had increased numbers of IL-25+ and IL-25R+ cells, and Lee et al. found increased IL-25 expression in CRS patient nasal polyp tissue [23,24]. To our knowledge, no studies have found a correlation between IL-25, and/or its receptor, and AR or AERD patient tissue.

Similar to IL-33, IL-25 is classically considered to be an epithelial cytokine; however, endothelial cells, eosinophils, Th2 cells, and mast cells have also been shown to secrete IL-25 [1,2,23]. IL-25 binds to the IL-25R receptor–a heterodimeric receptor consisting of IL-17RA and IL-17RB–which is expressed on the surface of ILC2s, Th2 cells, eosinophils, DCs, macrophages, and NKT cells [2]. The initial human ILC2 study that showed that IL-33 activates ILC2s also found that ILC2s in peripheral blood and fetal gut produce IL-13 in response to IL-25 stimulation [9]. Though IL-25 appears to serve a similar function to IL-33, experimental mouse models of asthma have elucidated some important differences. Mice demonstrate peak expression of IL-33 three hours post-allergen challenge, whereas IL-25 peaks, but to a lesser magnitude than IL-33, twelve hours post-challenge [25]. Additionally, IL-33 has been shown to be more potent than IL-25 in mouse models though relevance to humans in not clear [25]. Thus, because IL-25-induced activation of ILC2s may contribute to human asthma and CRSwNP pathogenesis, IL-25 represents a potential therapeutic target.

## Thymic Stromal Lymphopoietin (TSLP)

Thymic stromal lymphopoietin (TSLP) is an epithelial cytokine that was detected at increased levels in allergic airway disease well before ILC2s were discovered [26]. TSLP expression is increased in nasal polyps from CRS and AERD patients, AR patient nasal tissue, and in asthmatic airway epithelial cells (with levels positively correlating with disease severity) [14,27–29]. Additionally, TSLP SNPs that result in increased TSLP activity are associated with increased susceptibility to asthma [30].

Initially, TSLP was shown to prime DCs for adaptive Th2 responses and this role was thought to be primarily responsible for downstream type 2 inflammation driven by TSLP [26]. In addition to responding to TSLP, a recent human study showed that dendritic cells exposed to fungal allergen are also a potent source of TSLP [31]. Furthermore, recent studies have identified basophils and mast cells as additional sources of TSLP [32]. In humans, TSLP exists in two isoforms, long and short, which initiate inflammatory and homeostatic pathways, respectively [32]. Poposki et al. showed that TSLP fragments more potently activate type 2 immune responses compared to the full-length isoform [33]. TSLP exerts its effects by binding to the TSLPR and IL-7Ra heterodimeric receptor, which is expressed on ILC2s, DCs, and mast cells [33,34].

Aside from its known role in adaptive immunity, TSLP has been found to be a potent activator of ILC2s, independent of IL-33 [35]. In a landmark study, Mjosberg et al. showed that TSLP-stimulated ILC2s from peripheral blood and nasal polyp tissue upregulated GATA3, the master transcriptional regulator of type 2 cytokines, and produced IL-4, IL-5, IL-13 [36]. The effect of TSLP-driven activation of ILC2s was enhanced by addition of IL-33. Interestingly, a recent study by Liu et al. found that, of the epithelial cell-derived cytokines, only TSLP was able to induce ILC2 corticosteroid resistance (an effect also known to be present in mouse ILC2s) [37,38]. Importantly, an anti-TSLP antibody was also recently shown to significantly reduce airway eosinophils and hyperresponsiveness in asthmatics following allergen challenge [39]. Together, these results suggest that TSLP is a potent inducer of ILC2-driven type 2 inflammation as well as adaptive Th2 responses in humans; therefore, TSLP represents a therapeutic target for allergic airway diseases.

## Additional Cytokine Regulation of ILC2s

### IL-2 and IL-7

The development of ILC2s from common lymphoid progenitor cells requires IL-7R and IL-2R expression, and mice lacking IL-2R or IL-7R lack functional ILC2s [21,40]. IL-7 is a hematopoietic growth factor produced by epithelial cells and DCs that binds to IL-7R on ILC2s to promote homeostasis and proliferation [41]. IL-2 is produced by many lymphocytes and also promotes ILC2 homeostasis and proliferation by acting through the IL-2 receptor [41]. Though IL-2 and IL-7 are largely homeostatic for ILC2 responses, there is one report that demonstrated that IL-2 treatment of RAG1 deficient mice (lack B and T cells, but have ILC2s) led to increased ILC2 proliferation, activation, and dermatitis [42]. This suggests that, under specific conditions, IL-2 could be pro-inflammatory through ILC2 activation.

### IL-1 and IL-12

In 2016, two independent groups reported that IL-1 cytokines were capable of activating ILC2s and promoting ILC plasticity [43,44]. *In vitro*, IL-1α and IL-1β were found to potently induce human peripheral blood ILC2 proliferation and IL-5 and IL-13 production. Interestingly, IL-1β caused ILC2s to upregulate epithelial cell-derived cytokine receptors, while also priming ILC2s for conversion into ILC1s. These findings were confirmed *in vivo*, where the presence of IL-12 was the determining factor of whether IL-1β stimulation would result in ILC2 activation or ILC2 plasticity. The presence of IL-12 reduced ILC2 GATA3 expression and shifted IL-1β from an ILC2-activating cytokine to an ILC1-inducing cytokine.

### IL-4

IL-4 expression is increased in AERD, asthma, CRSwNP, CRSsNP, and AR [14,45,46]. Additionally, IL-4 SNPs are associated with increased asthma and AR risk [46]. ILC2s, mast cells, eosinophils, and basophils are capable of producing IL-4, and ILC2s also express the IL-4 receptor [2,43,47]. Thus, IL-4 can act in an autocrine fashion on ILC2s. Initial animal studies examining the relationship between IL-4 and ILC2s found that mouse lung ILC2s upregulated IL-9, IL-13, CCL3, CCL5, and CCL11 expression following IL-4 stimulation *in vitro*, and IL-33 potentiated this activation [48]. Interestingly, IL-5 was not upregulated with IL-4 alone, which suggests distinct activation mechanisms. Further, IL-4 alone was not sufficient to induce proliferation, but it did enhance IL-33-driven ILC2 proliferation. A recent human study found IL-4 to play a role similar to IL-2 and IL-7 in ILC2 maintenance [43]. Like prior animal studies, IL-4 was shown to potentiate IL-33-driven ILC2 proliferation and cytokine production; however, in contrast to the previous animal studies, IL-4 alone was not sufficient to induce cytokine production. Importantly, IL-4 was found to upregulate the expression of the PGD2 receptor CRTH2, which could then render ILC2s more responsive to PGD2. Furthermore, IL-4 prevented ILC2 to ILC1 conversion. Thus, IL-4 plays a unique role in ILC2 maintenance, proliferation, and activation. Multiple IL-4-inhibiting monoclonal antibody therapies have been tested in asthma clinical trials though the antibodies largely did not display efficacy. However, targeting IL-4R/IL-13R with dupilumab was recently FDA approved for asthma based on strong clinical trial data [49,50].

### TL1A

TL1A, a member of the TNF superfamily, has recently been shown to regulate mouse and human ILC2s. Activated T cells, myeloid cells, and endothelial cells are known sources of TL1A [51]. TL1A is the ligand for the DR3 receptor, which is expressed by ILC2s, T cells, and NKT cells [1,51,52]. Two recent studies reported that TL1A potently activates ILC2s [51,52]. Meylan et al. showed that mouse mesenteric lymph node (mLN) ILC2s stimulated *in vitro* with TL1A alone induced IL-5, IL-6, IL-13, and IL-17A secretion, and this effect was potentiated by IL-7. Interestingly, TL1A only induced IL-9 production when combined with IL-7. In contrast to their *in vitro* findings, exogenous TL1A was found to have no effect on ILC2 proliferation or cytokine production *in vivo*. Yu et al. confirmed Meylan et al.’s *in vitro* findings that murine mLN ILC2s stimulated with TL1A alone produce significantly more IL-5 and IL-13 compared to control. However, Yu et al.’s work showed that exogenous TL1A administration was sufficient to increase mouse ILC2 numbers and cytokine production *in vivo*. Additionally, the authors showed that TL1A alone was sufficient to induce human ILC2 IL-5 and IL-13 production and further potentiated IL-25 and IL-33-induced ILC2 cytokine production. Taken together, these findings suggest that TL1A could play a role in human allergic airway disease and represents a novel potential target for therapeutic intervention.

### IL-9

In 2011, Wilhelm et al. was the first to report that IL-9 maintains activated ILC2s through IL-9R [53]. In 2013, using a mouse helminth model of type 2 inflammation, the same group demonstrated that IL-9 potentiates ILC2 IL-5 and IL-13 production in an autocrine fashion [54]. Unlike IL-7, IL-9 was able to effectively maintain only activated ILC2s, rather than all ILC2s, and protect them from activation-induced apoptosis. Thus, IL-9 could be critical to sustaining type 2 inflammation through ongoing ILC2 activation. Additional studies are necessary to determine whether IL-9 has similar effects on ILC2 function in humans.

### Interferons & IL-27

A recent animal study reported that the Th1 cytokines IFN-γ and IL-27, and the type 1 interferon IFN-β, potently reduce ILC2 proliferation and cytokine production by directly targeting ILC2s [55]. Interestingly, the authors found that the ability of these cytokines to suppress ILC2 function was dependent upon localization: ILC2s from the mLN were sensitive to IL-27 suppression and resistant to IFN-γ, whereas tissue resident ILC2s were more sensitive to IFN-γ.

Furthermore, a recent report showed that IFN-β potently and directly suppressed mouse lung ILC2 proliferation and IL-5 production [56]. The group also demonstrated that purified human cord blood-derived ILC2s exposed to IFN-β had significantly reduced cytokine production and proliferation. Importantly, IFN SNPs are associated with asthma, and treatment of asthmatics with IFNs has been shown to reduce type 2 airway pathology.

### IL-10 and TGF-β

ILC2s highly express IL-10R and TGF-β receptors, and regulatory T cell (Treg) IL-10 and TGF-β production has recently been reported to directly inhibit IL-33-induced ILC2 IL-5 and IL-13 production [57,58]. Interestingly, the costimulatory ligand/receptor pair ICOS/ICOSL was required for full Treg mediated suppression, suggesting both direct (cell contact) and indirect (cytokines) dampening of ILC2s by Tregs. Additional studies are required to determine whether targeting Tregs during a natural Th2 disease course has an effect on ILC2s.

## Lipid Mediators that Regulate ILC2s

A plethora of cell types produce eicosanoids that, in turn, act on many different cell types to drive inflammation. In allergic airway disease pathophysiology, the primary producers of eicosanoids are activated mast cells, eosinophils, DCs, and macrophages [59]. The eicosanoid family members, prostaglandins (PGs) and leukotrienes (LTs), are bioactive lipid products of arachidonic acid (AA) metabolism. Two distinct metabolic pathways downstream of AA are the cyclooxygenase (COX) pathway, which produces PGs, and the 5-lipooxygenase (5LO) pathway, which produces LTs.

## Leukotrienes

The 5LO pathway of AA metabolism first produces LTA4, which can then be converted to the parent cysteinyl leukotriene (CysLT) LTC4 as well as LTB4. LTB4 is a final metabolite in its pathway, whereas LTC4 is rapidly converted into LTD4, and then into the terminal CysLT LTE4 [59].

## Cysteinyl Leukotrienes (CysLTs)

The CysLTs LTC4, LTD4, and LTE4 are elevated in asthma, allergic rhinitis, AERD, and CRS [59]. CysLTs were initially identified as a cause of bronchoconstriction after CysLT aerosol administration resulted in airway hyper responsiveness in both asthmatics and non-asthmatics [60]. Since then, several reports have established CysLTs in pro-inflammatory roles during type 2 immunity [61–63]. There are two primary CysLT receptors, CysLT1R and CysLT2R. CysLT1R and CysLT2R are capable of binding all of the CysLTs; however, CysLT1R binds LTD4 with the greatest affinity and has a low affinity for LTE4, while CysLT2R binds LTD4 and LTC4 with equal affinity and also has a low affinity for LTE4 [16]. Recently, additional receptors, P2Y12 and GPR99, were shown to mediate responses to LTE4 [64].

In 2013, Doherty et al. was the first to report that mouse lung ILC2s express CysLT1R and ILC2s are activated *in vitro* and *in vivo* by LTD4 [15]. CysLT-induced ILC2 activation and calcium influx was found to be dependent on CysLT1R as activation was abrogated by administration of montelukast, a CysLT1R antagonist. Interestingly, ILC2 activation driven by the fungal allergen *Alternaria* was potentiated by LTD4, which suggests IL-33 and LTD4 use non-redundant signaling mechanisms as *Alternaria* primarily mediates ILC2 responses through IL-33 [65]. Very recently, two studies confirmed that mouse lung ILC2s are activated, both *in vitro* and *in vivo*, by all of the CysLTs and that IL-33-induced ILC2 activation is further potentiated by CysLTs *in vitro* and *in vivo* [16,66].

The effect of CysLTs on human ILC2s appears to be conserved as Salimi et al. recently reported that: human ILC2s express CysLT1R, ILC2s are activated by CysLTs, and LTE4 potentiates IL-33 and IL-25 induced ILC2 activation in a CysLT1R-dependent manner [67,68]. Interestingly, a previous report showed that LTE4-induced ILC2 IL-5 production *in vivo* was not blocked by montelukast, though this could be due to differences between humans and mice and/or conditions (*in vivo vs. in vitro* ) [15]. Overall, CysLTs are potent activators of ILC2s, in both humans and mice, and are largely CysLT1R-dependent. While CysLT1R inhibition has long been used in the clinic to manage asthma, AR, CRS, and AERD symptoms, it is possible that combinational therapies blocking CysLTs and epithelial cell-derived cytokines will further alleviate type 2 cytokine-driven symptoms. Additional studies are needed to elucidate the function of the CysLT2, GPR99, and P2Y12 receptors in ILC2-driven allergic airway inflammation.

## Leukotriene B4 (LTB4)

LTB4 binds the LTB4R1 and LTB4R2 receptors. LTB4R1 is the primary receptor for LTB4 and is known to mediate chemotaxis whereas LTB4R2 binds LTB4, and other eicosanoids, with a relatively low affinity [69]. Recently, Moltke et al. reported high levels of LTB4R1, and not LTB4R2, expression on mouse lung ILC2s [16]. The LTB4R1 receptor was found to be functional as LTB4 stimulation *in vitro* increased ILC2 IL-13 production and was ameliorated in ILC2s lacking the LTB4R1 receptor. Interestingly, no effect of LTB4 was found *in vivo*. Additional studies are necessary to elucidate the role of LTB4 in human ILC2-driven allergic airway disease.

## Prostaglandins

The COX pathway of AA metabolism generates a wide variety of PGs. However, the PGs that will be discussed in this review are those that have been shown to regulate ILC2s, PGD2 and PGI2.

## Prostaglandin D2 (PGD2)

PGD2 expression is elevated in patients with severe asthma, CRS, AR, and is a dominant contributor to AERD pathology [14,70]. PGD2 is primarily synthesized by mast cells, but can also be produced by eosinophils [71]. Binding of PGD2 to CRTH2 expressed by ILC2s, Th2 cells, eosinophils, and basophils and results in type 2 cytokine production, chemotaxis, and increased cell survival [71].

In 2011, human ILC2s were first identified using the CRTH2 receptor, which distinguishes ILC2s from ILC1s and ILC3s [9]. A later study showed that human peripheral blood ILC2s produce increased IL-13 in response to PGD2 in the presence of IL-2, IL-25, and IL-33 [72]. PGD2 was subsequently found to induce ILC2 chemotaxis, an effect that was blocked by a CRTH2 inhibitor [71]. Xue et al. confirmed that PGD2 activates and induces chemotaxis of human ILC2s as well as potentiates the effect of IL-25 and IL-33 in the presence of IL-2 [67]. Aside from the classic ILC2 cytokines IL-4, IL-5, IL-13, the group also found additional pro-inflammatory cytokines produced by ILC2s including IL-3, IL-8, IL-9, IL-21, GM-CSF, and CSF1. Further, they showed that ILC2s up-regulated IL-25 and IL-33 receptors following PGD2 stimulation and hypothesized that PGD2 primed ILC2s for a potentiating effect by IL-25 and IL-33. In 2015, Wojno et al. showed that human ILC2 CRTH2 expression is tissue specific: peripheral blood ILC2s express higher levels of CRTH2 than lung ILC2s. Further, the group used a mouse model to show that CRTH2 regulates ILC2 chemotaxis *in vivo* [73]. The reduction of CRTH2 on human tissue ILC2s is an important finding as human ILC2s are identified by their CRTH2 expression. Therefore, studies of human tissue ILC2s may be excluding CRTH2-negative or -low populations when identifying ILC2s. Thus, it is vital that unique and constitutive identifying markers for ILC2s are discovered. Overall, these findings strongly demonstrate that PGD2 has multiple effects on ILC2s that could contribute to disease pathogenesis.

## Prostaglandin I2 (PGI2)

PGI2, another PG product of AA metabolism, has also been implicated in human allergic airway disease. Genome wide association studies previously showed that PGI2 SNPs are associated with aspirin intolerant asthma [74], and the PGI2 metabolite, 6-keto-PGF1α, was elevated in human lung tissue during *in vitro* anaphylaxis assays [75]. In the clinic, PGI2 analogs are commonly used to treat pulmonary hypertension; however, studies examining the therapeutic effect of PGI2 in human allergic airway disease have, thus far, been inconclusive. One study showed no change in FEV1 (expired lung volume in 1 second) and airway hyper responsiveness in response to methacholine challenge after PGI2 administration, while another study saw a decrease in FEV1 [76,77].

PGI2 is highly expressed in lung tissue and is produced by smooth muscle cells, endothelial cells, fibroblasts, follicular dendritic cells, and thymic nurse cells [76,78]. PGI2 has predominately anti-inflammatory properties and exerts its effects through binding of the prostacyclin receptor (IP), which is expressed by many immune cell types including bone marrow-derived DCs, Th1 cells, Th2 cells, and ILC2s [77]. PGI2-IP interactions ultimately result in smooth muscle relaxation, reduced cellular proliferation, and anti-inflammatory mechanisms including increased secretion of IL-10 by Tregs [78].

In 2016, Zhou et al. was the first to report that PGI2 inhibits ILC2 function [79]. In this study, bone marrow-derived ILC2s stimulated with IL-33 *in vitro* had reduced IL-5 and IL-13 production and proliferation in the presence of Cicaprost, a PGI2 analog. This attenuation was prevented in ILC2s lacking the IP receptor. Further, *Alternaria* -challenged IP knockout mice exhibited both increased numbers of lung ILC2s and increased ILC2 IL-5 and IL-13 production compared to wild type mice. Furthermore, wild type mice given both Cicaprost and *Alternaria* had lower levels of ILC2s and type 2 cytokine production, compared to mice given *Alternaria* alone. Finally, Cicaprost was found to inhibit IL-33-stimulated human peripheral blood ILC2 IL-5 and IL-13 production. These findings suggest that PGI2 might inhibit ILC2-driven allergic airway inflammation in humans.

## Specialized Pro-Resolving Lipid Mediators

Lipoxin A4 (LXA4) is generated by the 15-lipooxygenase (15LO) pathway in eosinophils, epithelial cells, and macrophages. Patients with severe asthma have reduced levels of LXA4 and its receptor, ALX/FPR2. A 1992 study found that patients given LXA4 following LTC4 challenge exhibited a reduction of asthmatic airway responses suggesting a protective role of LXA4 in asthma [70,80]. Recently, Barnig et al. found that human peripheral blood ILC2s express the LXA4 receptor and that LXA4 inhibited ILC2 responses in the presence of IL-25, IL-33, and PGD2 *in vitro* [72].

Maresin-1 is a product of omega-3 fatty acid metabolism. One report investigated the effects of maresin-1 and ILC2 responses [81]. Using a metabolipidomics approach, Krishnamoorthy et al. found that maresin-1 is increased in mouse lung tissue during the resolution phase of allergic inflammation. Maresin-1 administration *in vivo* inhibited ILC2 activation and promoted the generation of Tregs resulting in the resolution of inflammation. Additional studies are necessary to determine the role of maresin-1 in human ILC2 responses.

## ILC2 Regulation in Human Airway Disease

### Asthma

Asthma is a chronic lung disease characterized by airway inflammation, increased mucous production, hyperresponsiveness and remodeling, along with symptoms that include dyspnea, wheezing, chest tightness, and coughing [1,2]. Although there are many subtypes of asthma, a large portion of asthmatics have high levels of type 2 cytokines in their airways [82]. This suggests contributions from ILC2s in addition to CD4+ Th2 cells. Several pro-inflammatory cytokines (IL-25, IL-33 and TSLP) and lipid (CysLTs and PGD2) mediators are also elevated in asthmatic airways, which could drive ILC2 responses [18,23,27,59,70].

In 2013, the first report examining ILC2s and human asthma found no difference in peripheral blood ILC2 numbers between severe asthmatics, mild asthmatics, and healthy controls [72]. However, subsequent reports have shown differences in ILC2 numbers and activation between asthmatics and non-asthmatics. In 2014, Bartemes et al. found increased numbers of peripheral blood ILC2s and Th2 cytokine responses in allergic asthmatics compared to controls [3]. This finding was supported by a 2016 study that found increased numbers of activated ILC2s in the blood and sputum of severe asthmatics compared to mild, atopic asthmatics and healthy controls [4]. Furthermore, increased numbers of ILC2s have been found in both adult and pediatric asthma patients compared to controls [5,6]. Very recent studies have also demonstrated that ILC2s are increased after allergen challenge in asthmatics and that TSLP imparts ILC2 corticosteroid resistance in severe asthmatics [37,83]. The precise role of ILC2s in human asthma remains to be elucidated; however, these findings along with a multitude of animal studies suggest that ILC2s may contribute significantly to disease pathogenesis.

### Allergic rhinitis

Allergic rhinitis is caused by an IgE-mediated and type 2 inflammatory response to inhaled allergens which results in nasal symptoms including sneezing, itchiness, difficulty breathing, and discharge [84]. Previous work demonstrated that challenging sensitized AR patients with allergens causes a rapid increase in PGD2 production. Given that PGD2 has been shown to cause ILC2 activation, proliferation, and chemotaxis, it is plausible that ILC2s play a role in AR pathogenesis. In support of ILC2 activation in AR, IL-33 expression was found to be increased in the nasal secretions of patients with AR compared to control patients [85].

The first study to examine the relationship between AR and ILC2s found that nasal cat allergen challenge of cat-sensitized AR patients led to significantly increased percentage of ILC2s in peripheral blood compared to control challenge [7]. Consistent with this finding, a subsequent report found that peripheral blood ILC2s were increased in patients with grass pollen-sensitized AR during the pollen season compared to control patients, and that ILC2 levels were reduced by subcutaneous immunotherapy [8]. However, a separate report found that patients with AR have neither enhanced type 2 responses nor increased levels of ILC2s in the peripheral blood [3]. A recent study may shed light on these differences in results as subtypes of AR including House dust mite (HDM)- and mugwort-mediated AR were found to have differences in ILC2 responses [86]. The authors showed that peripheral blood ILC2s were increased, and had greater activation status, in patients with HDM-AR compared to mugwort-AR. The authors speculated that the differences could be due to the different mechanisms of action and/or levels of immunogenicity. Taken together, these findings demonstrate that ILC2s may promote specific subtypes of AR. Additional studies are needed to illuminate the overall role of ILC2s in this IgE driven nasal disorder.

### Chronic rhinosinusitis

Chronic rhinosinusitis is an inflammatory disease of the nasal and paranasal sinuses and is often associated with asthma and AR [2]. In some cases, CRS pathogenesis includes the development of nasal polyps (CRSwNP), which are classified as eosinophilic or non-eosinophilic. Nasal polyps from patients with CRS have elevated levels of CysLTs, IL-33, TSLP, and IL-4 [2,34] and multiple groups have shown that ILC2s are enriched in nasal polyps, especially eosinophilic polyps, compared to control tissue [9–12]. Together, these findings suggest that ILC2s may contribute to CRS pathogenesis and that eosinophilic polyposis is ILC2-driven.

## Aspirin Exacerbated Respiratory Disease (AERD)

AERD is a chronic inflammatory disease of the respiratory tract that includes moderate to severe asthma, nasal polyps, and respiratory reactions to COX-1 enzyme inhibitor medications [14]. Respiratory tissue from AERD patients has intense tissue eosinophilia, mast cell activation, and high levels of PGs (specifically PGD2) and LTs [14,87]. Recently, elevated levels of IL-4, TSLP, and IL-33 have also been found in patients with AERD [14,88,89]. The findings of increased PGD2, CysLTs, IL-33, and TSLP in AERD, as well as eosinophilic nasal polyps enriched in ILC2s, strongly suggest that ILC2s play a role in AERD pathogenesis. In 2017, Eastman et al. were the first to identify changes in ILC2s in AERD. Increased numbers of ILC2s were found in the nasal mucosa, and decreased numbers of ILC2s in the peripheral blood, during aspirin (COX-1 inhibitor) challenge in AERD patients [13]. One possible explanation for this is that ILC2s migrate from the peripheral blood to the nasal mucosa during reactions in response to PGD2 interacting with CRTH2 on ILC2s. Additional studies are necessary to elucidate the functional role, and possible mechanism of migration, of ILC2s in AERD.

## Summary

In the last decade since ILC2s have been discovered, a large number of cytokines and lipid mediators have been shown to positively and negatively regulate ILC2 homeostasis, proliferation, and activation in both mouse models of airway disease and in human samples. ILC2s and many positive regulators including CysLTs, PGD2, IL-33 and TSLP are elevated in the airways of patients with allergic diseases, which supports an environment of ILC2-driven inflammation. Development of therapies that target specific mediators upstream of ILC2s are underway and the challenge of uncovering ILC2-specific therapy remains. However, based on the discovery of negative regulators of ILC2s and finding that combinations of specific mediators can potentiate ILC2-driven inflammation, it is plausible that combinational therapies could better treat allergic airway disease and improve the quality of life of patients.
